# Social Determinants of Health Affect Psychological Distress among People with Disabilities

**DOI:** 10.3390/ijerph21101359

**Published:** 2024-10-15

**Authors:** Jessica Kersey, Amie Devlin, Sarah Shyres, Emily A. Kringle, Ashley J. Housten

**Affiliations:** 1 Program in Occupational Therapy, School of Medicine, Washington University, St. Louis, MO 63110, USA; shyres@wustl.edu; 2 Independent Researcher, St. Louis, MO 63118, USA; 3 School of Kinesiology, College of Education and Human Development, University of Minnesota, Minneapolis, MN 55455, USA; ekringle@umn.edu; 4 Department of Surgery, School of Medicine, Washington University, St. Louis, MO 63110, USA; ahousten@wustl.edu

**Keywords:** social determinants of health, persons with disabilities, neighborhood characteristics, community resources

## Abstract

People with disabilities experience inequitable exposure to social determinants of health (SDOH) that contribute to disparate health outcomes, including psychological distress. There is little research examining which SDOH have the strongest effect on psychological distress among people with disabilities. This leaves healthcare providers and policy makers with insufficient information to make well-informed treatment decisions or allocate resources effectively. We explored the association between SDOH and disability and which factors may moderate the association between disability and psychological distress. Using data from the US Census Bureau’s Household Pulse Survey (Phase 3.5), we examined SDOH among people with and without disability (*n* = 26,354). Among people with disability, the odds of severe psychological distress were highest among those who had low incomes (OR = 4.41, 95% CI: 3.51–5.60), were food insecure (OR = 3.75, 95% CI: 3.43–4.10), housing insecure (OR = 3.17, 95% CI: 2.82–3.58), or were unable to work (OR = 1.98, 95% CI: 1.80–2.18). Only difficulty paying for household expenses moderated the association between disability and severe psychological distress (OR = 9.81, 95% CI: 7.11–13.64). These findings suggest that supporting employment and economic opportunities and improving access to safe and affordable housing and food may improve psychological well-being among people with disabilities.

## 1. Introduction

Disability is prevalent in the United States. Recent estimates suggest that 67.2 million Americans, or 26.8% of the US adult population, has some degree of disability [1]. Biopsychosocial models of disability suggest that disability results from both the influences of a health condition on bodily functions and environmental influences that affect participation in daily life [2]. While the importance of the environment—including social networks, accessible spaces, and access to services and resources—has been acknowledged, most interventions that aim to maximize quality of life among people with disabilities focus on the heath condition and the resulting physical, emotional, or cognitive impairments [3]. Alternatively, there have been few community or social-level interventions. This may be because few studies have examined which specific environmental factors are the most strongly associated with quality of life among people with disabilities.

Social determinants of health (SDOH) comprise the environmental and social factors that affect health and can be used to classify and examine environmental influences on disability. SDOH are defined by Healthy People 2030 as “the conditions of the environment where people are born, live, learn, work, play, worship, and age that affect a wide range of health, functioning, and quality of life outcomes and risks” [4]. These factors fall into five domains: economic stability, education access and quality, health care access and quality, the neighborhood and the built environment, and the social and community context. The Healthy People 2030 SDOH framework aligns with the limited research examining environmental influences on disability, thus representing a strong starting point for further investigation [5].

Existing research suggests that there are disparities in education, income, and healthcare access and use among people with disabilities [6,7,8,9]. However, these studies examined limited SDOH variables and had small sample sizes. Furthermore, these studies were not designed to examine the association between these select SDOH factors and health outcomes. Very few studies have examined SDOH that affect the relationship between disability and psychological distress. This leaves researchers, practitioners, and policy makers with a limited understanding of the most potent targets for policy or intervention that will have the greatest impact on quality of life among people with disabilities.

Furthermore, there is limited research examining exposure to SDOH among people with disabilities within current societal structures given the changes in policy and communities resulting from the COVID-19 pandemic. While several important changes may have been supportive of people with disabilities (e.g., expanded access to remote work and telehealth, distribution of stimulus funds), others had negative and disproportionate effects on people with disabilities (such as higher rates of unemployment and social isolation and inaccessible technologies and information) [10,11,12,13]. The few publications outlining SDOH among people with disabilities have examined data that were collected prior to the pandemic and thus may not represent current experiences due to changes in services, resources, risks, and benefits [7,8,14]. People with disabilities are at higher risk of serious illness and death related to COVID-19 than those without disabilities, and they continue to adjust their behaviors accordingly [15,16,17]. To make informed policy recommendations and healthcare decisions, it is important to understand exposure to the detrimental factors of SDOH in the current environment.

The purpose of this study was to identify associations between SDOH and well-being among people with disabilities and to identify SDOH that may moderate the relationship between disability and well-being.

## 2. Methods and Materials

### 2.1. Study Data

We used data from Phase 3.5 of the US Census Bureau’s Household Pulse Survey, a nationally representative survey of adults conducted between 27 July and 8 August 2022 [18,19]. This phase of the Household Pulse Survey contained data from a timeframe when the societal impacts of the COVID-19 pandemic were stabilizing but before the conclusion of the formal public health emergency. Later phases of data collection lacked key SDOH variables, making Phase 3.5 the most useful subset of data for answering the research question. We first examined the association between disability and psychological distress, and then we investigated SDOH variables that may moderate that association. Finally, analyzing data only from participants with a disability, we examined the association between SDOH variables and psychological distress.

### 2.2. Demographic and SDOH Variables

SDOH variables of interest were aligned with the Healthy People 2030 SDOH domains, described below.

- *Economic stability* variables included employment status, difficulty paying for expenses, food security, and housing security. Employment status was initially classified as a binary variable (presence vs. absence of work for pay in the last week) and then further classified as either not wanting to work (i.e., did not want to be employed at this time or retired) or not being able to work (i.e., sick/disabled, concerned about getting sick, or caring for children/elderly). Participants were classified as food insecure if they reported one of the following: (1) enough to eat, but not always the kinds of food they wanted to eat; (2) sometimes not enough to eat; and (3) often not enough to eat. To further examine access to affordable foods, we also examined whether participants had access to the Supplemental Nutrition and Assistance Program (SNAP), previously known as food stamps, which provides a monthly financial supplement to low-income families that must be spent on qualifying grocery items. Participants were classified as housing insecure if they indicated either of the following: (1) household not currently caught up on rent/mortgage payments or (2) eviction/foreclosure in the next two months was very/somewhat likely.
- *Education access and quality* included variables examining participants’ highest level of education and changes to education plans due to the pandemic.
- *Healthcare access and quality* variables included coverage by public health insurance and access to telehealth services for both adults and children within the household.
- *Neighborhood and built environment* variables included use of public transportation and access to food assistance, including food pantries/food banks, churches, or other sources of free meals or groceries.
- *Social and community context* variables included marital status and childcare availability. We included age, race, ethnicity, and gender as demographic covariates.

### 2.3. Disability

Disability status was classified based on the following self-reported difficulties: seeing (even while wearing glasses); hearing (even when using a hearing aid); remembering or concentrating; walking or climbing stairs; communicating in their usual language; and self-care (such as washing all over or dressing). Participants were classified as disabled if they reported difficulty in any of these domains [20].

### 2.4. Psychological Distress

Psychological distress was defined as symptoms of anxiety and depression reported on the Patient Health Questionnaire 4 (PHQ4) [21]. The PHQ-4 is a valid tool for measuring anxiety and depression, and it has established reliability (α = 0.92) [22]. It has a possible point range of 0–12, and higher scores indicate greater psychological distress. Our primary analysis included participants with moderate psychological distress (defined as a score ≥ 6 on the PHQ4), and our secondary analysis examined participants with severe psychological distress (defined as a PHQ4 score ≥ 9).

### 2.5. Statistical Methods

Participants with missing data related to disability, psychological distress, or SDOH variables of interest were removed from the analysis. To determine which SDOH and demographic variables to include in the multivariate analysis, we first conducted bivariate logistic regression to examine the association between demographic and SDOH variables and the odds of having a disability. We then selected variables to include in the analyses based on the results of the bivariate analyses and documented importance in the disability literature. We then used multivariate logistic regression to assess the association between SDOH variables and disability with psychological distress. Next, we assessed whether SDOH variables may moderate the association between disability and psychological distress. We used bivariate logistic regression to assess the association between SDOH variables and psychological distress among participants with a disability. Continuous variables were summarized using the mean, standard deviation (SD), the median, and the interquartile range (Q1, Q3). Categorical variables were summarized using frequency counts and percentages. Odds ratios (ORs) and 95% confidence intervals (95% CI) were reported for all logistic regressions.

## 3. Results

### 3.1. Overall Study Population

Of the 26,354 participants included in the analysis, 10,223 (38.8%) reported having a disability. The study population, described in Table 1, was highly educated (67.9% had an Associate’s degree or more). While females accounted for 52.1% of the non-disabled population, 60.8% of the disabled population was female. Participants in the overall sample had a mean (SD) age of 48.6 (14.9) years, although participants with a disability were slightly older (50.2; SD 15.3) than participants without a disability (46.0; SD 13.9). The vast majority were white (83.0%) and non-Hispanic (91.9%); these distributions were similar across those with and without disabilities.

Table 2 describes the univariate associations between individual demographic/SDOH variables and having a disability. In the unadjusted model, females had higher odds of having a disability than males (1.49 [1.41–1.56]), as did those who were transgender or another gender, although the sample sizes were small. Hispanic participants had higher odds of having a disability compared with non-Hispanic participants (1.14 [1.05–1.25]). Non-married participants had higher odds of having a disability than married participants (widowed: 2.81 [2.40–3.30]; divorced: 2.03 [1.88–2.19]; separated: 2.41 [1.93–3.03]; never married: 1.19 [1.12–1.27]). Income was inversely associated with having a disability. The odds of having a disability increased as the income category decreased. Similarly, participants who were housing insecure (2.33 [2.07–2.63]) or food insecure (3.14 [2.96–3.33]) had higher odds of having a disability relative to those who were not. Participants who did not work in the last week had higher odds of being disabled than those who had worked (1.86 [1.76–1.97]).

### 3.2. Association between Disability and Psychological Distress

In multivariable models (Table 3), participants with a disability had higher odds of moderate psychological distress (3.96 [3.65–4.31]) relative to those without a disability. This odds ratio increased when examining the odds of severe psychological distress (4.29 [3.80–4.86]). When examining the effect of the interaction between disability and demographic and SDOH variables on moderate psychological distress, there was no significant interaction between disability and any of the included variables (age, gender, marital status, employment in the past week, difficulty paying for expenses, food insecurity, or housing insecurity). When examining the effect of this interaction on severe psychological distress, there was a significant interaction between disability and difficulty paying for expenses (Table 4).

### 3.3. Participants with a Disability

Table 5 presents the odds of moderate and severe psychological distress among participants with a disability.

*Moderate Psychological Distress.* Among participants with a disability, those with moderate psychological distress were younger than those without (45.5 [14.0] vs. 52.5 [15.4]) and had slightly lower education levels (57.1% vs. 65.8% had an Associate’s degree or higher). Participants with moderate psychological distress were also less likely to be married than those without (41.5% vs. 55.1%). Participants who were Hispanic, Latino, or of Spanish origin had higher odds of moderate psychological distress (1.27 [1.33–1.41]) than those who were not, as did females (1.36 [1.26–1.46]), transgender participants (4.14 [2.93–5.90]), and those of another gender (2.41 [1.85–3.13]).

While there was no significant difference in the odds of moderate psychological distress by employment status, there was a difference based on whether participants did not want to work (0.44 [0.40–0.49]) or were not able to work (1.75 [1.61–1.91]) compared with participants who were working. Income was inversely related to moderate psychological distress, as were housing security (2.95 [2.63–3.32]) and food security (3.37 [3.14–3.60]).

*Severe Psychological Distress.* Females (1.26 [1.15–1.38]), those who identified as transgender (3.13 [2.17–4.47]), and those who identified as another gender (2.91 [2.19–3.84]) had higher odds of severe psychological distress relative to males. Again, income was inversely associated with severe psychological distress, as were housing security (3.17 [2.80–3.58]) and food security (3.75 [3.43–4.10). Difficulty paying for expenses was directly associated with odds of severe psychological distress compared to those who reported that paying for household expenses was not difficult at all. The odds of severe psychological distress increased with each difficulty level (a little difficult: 1.98 [1.70–2.12]; somewhat difficult: 3.96 [3.42–4.59]; very difficult: 11.63 [10.12–13.41]). Participants who did not work in the past week had higher odds of severe psychological distress (1.14 [1.04–1.24]) than those who did work in the past week; again, these odds varied depending on whether participants did not want to work (0.42 [0.36–0.48]) or were unable to work (1.98 [1.80–2.18]).

## 4. Discussion

This study examined SDOH among people with disabilities, and, furthermore, the SDOH that may moderate the association between disability and psychological distress. Among people with disabilities, those who were involuntarily unemployed, had lower incomes, were housing insecure, or were food insecure were most likely to experience psychological distress. The odds of psychological distress were high for those facing difficulties paying household expenses and who often went without enough food. These findings align with previous research demonstrating that people with disabilities experience disproportionately lower household income both in the United States [7,8,9,14] and globally [23,24,25]. Our findings further demonstrate the importance of food and housing security for their psychological well-being.

Despite high odds ratios between numerous SDOH and psychological distress among participants with disabilities, only difficulties paying for household expenses moderated the association between disability and severe psychological distress. Both housing security and food security had strong associations with psychological distress among disabled participants. It is possible that difficulty paying for “household expenses” is a more comprehensive variable describing overall economic burden and therefore moderated this relationship. Other research suggests that among low-income families, food, housing, and transportation costs are prioritized over other household expenses, and that debt, medical bills, and other expenses more often go unpaid [26,27]. The hardships associated with these difficulties and the inability to pay for additional comforts or self-care may affect the relationship between disability and psychological distress. 

These findings suggest that strengthening disability benefits, including systems to ensure safe and affordable housing and reliable sources of food, may improve psychological well-being among people with disabilities. While a full analysis of the most potent policy priorities to reduce housing instability and food insecurity are outside of the scope of this paper, prior research suggests some top policy priorities. These policy priorities include expanding access to housing/rental vouchers and priority access given to people with disabilities [28], expanding the restrictive eligibility criteria for SNAP for people with disabilities [29], and expanding Social Security Income and Social Security Disability Income [30]. Moreover, research has demonstrated that community-level food interventions (food banks, community kitchens) are promising but often demonstrate mixed results due to issues including stigma and limited locations/hours of operation [29,31,32]. This may suggest that providing food support through disability support organizations (e.g., Centers for Independent Living) may also alleviate food insecurity and improve well-being. Disability support organizations may consider partnering with local food banks or community kitchens to offer options on-site in a safe and supportive environment, and, furthermore, they may consider allocating more personnel to aid in navigating processes for obtaining housing vouchers, SNAP, and Social Security benefits.

These findings also suggest that there is an unmet need for mental health services among people with disabilities, who experience high rates of psychological distress. It has long been documented that mental health services are under-utilized among people with disabilities due to a wide range of barriers [33,34,35]. New research demonstrates that these barriers have been exacerbated since the start of the pandemic [10,36,37]. The present study demonstrates that disparities in psychological distress among people with disabilities persist. In addition to addressing SDOH to improve psychological well-being among people with disabilities, there remains a critical need at the policy level to expand accessible, high-quality mental health services through expanded insurance coverage, multi-level strategies to grow the mental health workforce, and focused efforts to ensure the physical and cognitive accessibility of mental health services for people with disabilities.

An important consideration in the interpretation of these results is the context of the COVID-19 pandemic. This study used data collected in July and August of 2022, when the most severe waves of COVID-19 were concluded, vaccinations and home test kits were no longer in short supply, and nearly all public mitigation strategies (stay at home orders, mask mandates, and vaccination mandates) had ended. This was a time of beginning to transition to a “new normal” in US society. However, very high rates of disability continue to be reported, particularly in the domains of cognitive and mobility disabilities. A full understanding of this phenomenon and its relationship to SDOH cannot be derived from this limited cross-sectional analysis.

This study has important limitations to consider. First, while this dataset included many SDOH variables, the range of these variables was still limited. Of the five categories of SDOH outlined by Healthy People 2030, some categories were covered more comprehensively than others. In particular, there is limited information about the effect of social networks, social support, and community-level supports. Other research has demonstrated the importance of these factors to mental and physical health, and it is possible that these would have been meaningful variables affecting the association between disability and psychological distress [7,17,38,39]. This study also used data from a limited time frame. The US Census Bureau constantly updated the survey questions across phases to address changing societal needs. The optimal ranges of SDOH, disability, and psychological distress variables were only available in a few phases of data collection. Thus, we are unable to examine whether these findings have remained stable over time or changed as we have progressed further from the peak of the COVID-19 pandemic. Finally, the sample in this study was not representative of the US population. This sample was highly educated, and racial and ethnic minority groups were under-represented. This work could be replicated in future research in samples representative of evolving US socio-demographics.

## 5. Conclusions

In conclusion, this study aimed to identify which SDOH variables may moderate the association between disability and psychological distress. Disabled participants who were unemployed, food or housing insecure, and had lower incomes had the highest odds of experiencing psychological distress. Difficulty paying for household expenses moderated the relationship between disability and severe psychological distress. These findings suggest the need for policy to better address the economic and financial needs of people with disabilities, with a focus on supporting housing needs and ensuring access to affordable or free food. These findings further suggest a critical, ongoing need to address the unmet mental health needs of people with disabilities.

## Figures and Tables

**Table 1:** Participant demographics and SDOH.

	Overall (26,354)	No Disability (10,223; 38.8%)	Disability (16,131; 61.2%)
**Demographic Characteristics and Social Determinants of Health**			
**Age (years)**			
Mean (SD)	48.6 (14.9)	46.0 (13.9)	50.2 (15.3)
Median [min, max]	47.0 [19.0, 89.0]	43.0 [19.0, 89.0]	50.0 [19.0, 89.0]
**Hispanic, Latino, or Spanish origin**			
No	24,079 (91.4%)	9406 (92.0%)	14,673 (91.0%)
Yes	2275 (8.6%)	817 (8.0%)	1458 (9.0%)
**Race**			
White alone	21,675 (82.2%)	8363 (81.8%)	13,312 (82.5%)
Black alone	2028 (7.7%)	760 (7.4%)	1268 (7.9%)
Asian alone	1204 (4.6%)	646 (6.3%)	558 (3.5%)
Any other race alone or race in combination	1447 (5.5%)	454 (4.4%)	993 (6.2%)
**Highest degree or level of school completed**			
Less than high school	119 (0.5%)	36 (0.4%)	83 (0.5%)
Some high school	295 (1.1%)	72 (0.7%)	223 (1.4%)
High school graduate or equivalent	2625 (10.0%)	773 (7.6%)	1852 (11.5%)
Some college, no degree/in progress	5408 (20.5%)	1601 (15.7%)	3807 (23.6%)
Associate’s degree (e.g., AA, AS)	2764 (10.5%)	854 (8.4%)	1910 (11.8%)
Bachelor’s degree (e.g., BA, BS, AB)	7860 (29.8%)	3413 (33.4%)	4447 (27.6%)
Graduate degree (e.g., master’s, professional, doctorate)	7283 (27.6%)	3474 (34.0%)	3809 (23.6%)
**Marital status**			
Now married	14,464 (54.9%)	6281 (61.4%)	8183 (50.7%)
Widowed	946 (3.6%)	203 (2.0%)	743 (4.6%)
Divorced	4114 (15.6%)	1129 (11.0%)	2985 (18.5%)
Separated	422 (1.6%)	102 (1.0%)	320 (2.0%)
Never married	6408 (24.3%)	2508 (24.5%)	3900 (24.2%)
**Gender**			
Male	10,752 (40.8%)	4802 (47.0%)	5950 (36.9%)
Female	15,133 (57.4%)	5325 (52.1%)	9808 (60.8%)
Transgender	150 (0.6%)	14 (0.1%)	136 (0.8%)
None of these	319 (1.2%)	82 (0.8%)	237 (1.5%)
**2021 household income before taxes**			
Less than USD 25,000	2724 (10.3%)	544 (5.3%)	2180 (13.5%)
USD 25,000–34,999	2128 (8.1%)	557 (5.4%)	1571 (9.7%)
USD 35,000–49,999	2729 (10.4%)	829 (8.1%)	1900 (11.8%)
USD 50,000–74,999	4425 (16.8%)	1491 (14.6%)	2934 (18.2%)
USD 75,000–99,999	3831 (14.5%)	1539 (15.1%)	2292 (14.2%)
USD 100,000–149,999	5007 (19.0%)	2200 (21.5%)	2807 (17.4%)
USD 150,000–199,999	2573 (9.8%)	1291 (12.6%)	1282 (7.9%)
USD 200,000 and above	2937 (11.1%)	1772 (17.3%)	1165 (7.2%)
**Covered by public or private health insurance**			
Yes	25,014 (94.9%)	9778 (95.6%)	15,236 (94.5%)
No	1340 (5.1%)	445 (4.4%)	895 (5.5%)
**Work for either pay or profit in the last 7 days**			
Yes	18,516 (70.3%)	7964 (77.9%)	10,552 (65.4%)
No	7838 (29.7%)	2259 (22.1%)	5579 (34.6%)
Not working: did not want to work ^a^	3756 (14.3%)	1086 (10.6%)	2670 (16.6%)
Not working: not able to work	4082 (15.5%)	1173 (11.5%)	2909 (18.0%)
**Difficulty paying for household expenses in the last 7 days**			
Not at all difficult	10,554 (40.0%)	5614 (54.9%)	4940 (30.6%)
A little difficult	6887 (26.1%)	2473 (24.2%)	4414 (27.4%)
Somewhat difficult	5046 (19.1%)	1353 (13.2%)	3693 (22.9%)
Very difficult	3867 (14.7%)	783 (7.7%)	3084 (19.1%)
**Food Insecurity ^b^**			
Food insecure	8769 (33.3%)	1938 (19.0%)	6831 (42.3%)
Not food insecure	17,585 (66.7%)	8285 (81.0%)	9300 (57.7%)
Enough of the kinds of food I/we wanted to eat	17,585 (66.7%)	8285 (81.0%)	9300 (57.7%)
Enough, but not always the kinds of food I/we wanted to eat	6812 (25.8%)	1643 (16.1%)	5169 (32.0%)
Sometimes not enough to eat	1500 (5.7%)	236 (2.3%)	1264 (7.8%)
Often not enough to eat	457 (1.7%)	59 (0.6%)	398 (2.5%)
**Free groceries/meals or Supplemental Nutrition Assistance Program (SNAP) within the last 7 days**			
Did not receive SNAP and/or other free groceries/meals	23,492 (89.1%)	9606 (94.0%)	13,886 (86.1%)
Received SNAP or other free groceries/meals	2862 (10.9%)	617 (6.0%)	2245 (13.9%)
**Free groceries from a food pantry, food bank, church, or other place that helps with free food**			
Yes	1055 (4.0%)	202 (2.0%)	853 (5.3%)
No	25,299 (96.0%)	10,021 (98.0%)	15,278 (94.7%)
**Received benefits from the Supplemental Nutrition Assistance Program (SNAP) within the last 7 days**			
Yes	2231 (8.5%)	478 (4.7%)	1753 (10.9%)
No	24,123 (91.5%)	9745 (95.3%)	14,378 (89.1%)
**Housing insecurity ^c^**			
Housing insecure	1616 (6.1%)	358 (3.5%)	1258 (7.8%)
Not housing insecure	24,738 (93.9%)	9865 (96.5%)	14,873 (92.2%)
**Disability**			
**Difficulty seeing, even when wearing glasses**			
No	18,130 (68.8%)	10,223 (100%)	7907 (49.0%)
Yes	8224 (31.2%)	0 (0%)	8224 (51.0%)
**Difficulty hearing, even when using a hearing aid**			
No	21,813 (82.8%)	10,223 (100%)	11,590 (71.8%)
Yes	4541 (17.2%)	0 (0%)	4541 (28.2%)
**Difficulty walking or climbing stairs**			
No	20,488 (77.7%)	10,223 (100%)	10,265 (63.6%)
Yes	5866 (22.3%)	0 (0%)	5866 (36.4%)
**Difficulty remembering or concentrating**			
No	15,212 (57.7%)	10,223 (100%)	4989 (30.9%)
Yes	11,142 (42.3%)	0 (0%)	9358 (69.1%)
**Difficulty with self-care, such as washing all over or dressing**			
No	24,446 (92.8%)	10,223 (100%)	14,223 (88.2%)
Yes	1908 (7.2%)	0 (0%)	1908 (11.8%)
**Difficulty communicating; for example, understanding or being understood in their primary language**			
No	24,565 (93.2%)	10,223 (100%)	14,342 (88.9%)
Yes	1789 (6.8%)	0 (0%)	1613 (11.1%)
**Psychological Distress**			
**Total PHQ4 score**			
Mean (SD)	3.39 (3.52)	1.84 (2.55)	4.36 (3.70)
Median [min, max]	2.00 [0, 12.0]	1.00 [0, 12.0]	4.00 [0, 12.0]

^a^ Not wanting to work was defined as did not want to be employed at this time or retired. ^b^ Food insecurity was defined as enough to eat but not always the kinds of food I/we wanted to eat, sometimes not enough to eat, or often not enough to eat. ^c^ Housing insecure was defined as either rent/mortgage not being current or eviction/foreclosure being somewhat/very likely.

**Table 2:** Odds of disability by demographic factors and SDOH (univariate model).

	No Disability (10,223; 38.8%)	Disability (16,131; 61.2%)	Odds Ratio (Odds of Disability)	95% Confidence Interval
**Age (years)**				
Mean (SD)	46.0 (13.9)	50.2 (15.3)	1.020 *	1.02–1.02
Median [min, max]	43.0 [19.0, 89.0]	50.0 [19.0, 89.0]		
**Hispanic, Latino, or Spanish origin**				
No	9406 (92.0%)	14,673 (91.0%)	REF	
Yes	817 (8.0%)	1458 (9.0%)	1.144 *	1.05–1.25
**Race**				
White alone	8363 (81.8%)	13,312 (82.5%)	REF	
Black alone	760 (7.4%)	1268 (7.9%)	1.048	0.95–1.15
Asian alone	646 (6.3%)	558 (3.5%)	0.543 *	0.48–0.61
Any other race alone or race in combination	454 (4.4%)	993 (6.2%)	1.374 *	1.23–1.54
**Highest degree or level of school completed**				
Less than high school	36 (0.4%)	83 (0.5%)	REF	
Some high school	72 (0.7%)	223 (1.4%)	1.343	0.83–2.15
High school graduate or equivalent	773 (7.6%)	1852 (11.5%)	1.039	0.69–1.54
Some college, no degree/in progress	1601 (15.7%)	3807 (23.6%)	1.031	0.69–1.52
Associate’s degree (e.g., AA, AS)	854 (8.4%)	1910 (11.8%)	0.970	0.64–1.43
Bachelor’s degree (e.g., BA, BS, AB)	3413 (33.4%)	4447 (27.6%)	0.565 *	0.38–0.83
Graduate degree (e.g., master’s, professional, doctorate)	3474 (34.0%)	3809 (23.6%)	0.476 *	0.32–0.70
**Marital status**				
Now married	6281 (61.4%)	8183 (50.7%)	REF	REF
Widowed	203 (2.0%)	743 (4.6%)	2.809 *	2.40–3.30
Divorced	1129 (11.0%)	2985 (18.5%)	2.029 *	1.88–2.19
Separated	102 (1.0%)	320 (2.0%)	2.408 *	1.93–2.19
Never married	2508 (24.5%)	3900 (24.2%)	1.194 *	1.12–1.27
**Gender**				
Male	4802 (47.0%)	5950 (36.9%)	REF	REF
Female	5325 (52.1%)	9808 (60.8%)	1.487 *	1.41–1.56
Transgender	14 (0.1%)	136 (0.8%)	7.840 *	4.69–14.24
None of these	82 (0.8%)	237 (1.5%)	2.333	1.82–3.02
**2021 household income before taxes**				
Less than USD 25,000	544 (5.3%)	2180 (13.5%)	6.095 *	1.41–1.56
USD 25,000–34,999	557 (5.4%)	1571 (9.7%)	4.290 *	3.80–4.85
USD 35,000–49,999	829 (8.1%)	1900 (11.8%)	3.486 *	3.12–3.89
USD 50,000–74,999	1491 (14.6%)	2934 (18.2%)	2.993 *	2.72–3.30
USD 75,000–99,999	1539 (15.1%)	2292 (14.2%)	2.265 *	2.05–2.50
USD 100,000–149,999	2200 (21.5%)	2807 (17.4%)	1.941 *	1.77–2.13
USD 150,000–199,999	1291 (12.6%)	1282 (7.9%)	1.510*	1.36–1.68
USD 200,000 and above	1772 (17.3%)	1165 (7.2%)	REF	REF
**Loss of employment income in the last 4 weeks**				
Yes	657 (6.4%)	2015 (12.5%)	REF	REF
No	9566 (93.6%)	14,116 (87.5%)	2.078 *	1.90–2.28
**Work for either pay or profit in the last 7 days**				
Yes	7964 (77.9%)	10,552 (65.4%)	REF	REF
No	2259 (22.1%)	5579 (34.6%)	1.864 *	1.76–1.97
Not working: did not want to work ^a^	1086 (10.6%)	2670 (16.6%)	1.856 *	1.72–2.00
Not working: was not able to work	1173 (11.5%)	2909 (18.0%)	1.872 *	1.74–2.02
**Difficulty paying usual household expenses**				
Not at all difficult	5614 (54.9%)	4940 (30.6%)	REF	REF
A little difficult	2473 (24.2%)	4414 (27.4%)	2.028 *	1.90–2.16
Somewhat difficult	1353 (13.2%)	3693 (22.9%)	3.102 *	2.88–3.34
Very difficult	783 (7.7%)	3084 (19.1%)	4.476 *	4.10–4.89
**Food insecurity ^b^**				
Food insecure	1938 (19.0%)	6831 (42.3%)	3.140 *	2.96–3.33
Not food insecure	8285 (81.0%)	9300 (57.7%)	REF	REF
Enough of the kinds of food I/we wanted to eat	8285 (81.0%)	9300 (57.7%)	REF	REF
Enough, but not always the kinds of food I/we wanted to eat	1643 (16.1%)	5169 (32.0%)	2.803 *	2.63–2.99
Sometimes not enough to eat	236 (2.3%)	1264 (7.8%)	4.771 *	4.15–5.51
Often not enough to eat	59 (0.6%)	398 (2.5%)	6.010 *	4.60–7.99
**Free groceries/meals or Supplemental Nutrition Assistance Program (SNAP) within the last 7 days**				
Did not receive SNAP and/or other free groceries/meals	9606 (94.0%)	13,886 (86.1%)	REF	REF
Received SNAP or other free groceries/meals	617 (6.0%)	2245 (13.9%)	2.517 *	2.30–2.76
**Free groceries from a food pantry, food bank, church, or other place that helps with free food**				
Yes	202 (2.0%)	853 (5.3%)	REF	REF
No	10,021 (98.0%)	15,278 (94.7%)	2.770 *	2.38–3.24
**Received benefits from the Supplemental Nutrition Assistance Program (SNAP) within the last 7 days**				
Yes	478 (4.7%)	1753 (10.9%)	REF	REF
No	9745 (95.3%)	14,378 (89.1%)	2.486 *	2.24–2.76
**Housing insecure ^c^**				
Housing insecure	358 (3.5%)	1258 (7.8%)	2.331 *	2.07–2.63
Not housing insecure	9865 (96.5%)	14,873 (92.2%)	REF	REF

* *p* < 0.01; REF: reference group. ^a^ Not wanting to work was defined as did not want to be employed at this time or retired. ^b^ Food insecurity was defined as enough to eat but not always the kinds of food I/we wanted to eat, sometimes not enough to eat, or often not enough to eat. ^c^ Housing insecure was defined as either rent/mortgage not being current or eviction/foreclosure being somewhat/very likely.

**Table 3:** Odds of psychological distress (multivariable model).

	OR (Odds of Moderate Psychological Distress)	95% Confidence Interval	OR (Odds of Severe Psychological Distress)	95% Confidence Interval
**Disability**				
No	REF		*REF*	
Yes	3.96 *	3.65–4.31	4.29 *	3.80–4.86
**Age**		0.97–0.97	0.97 *	0.97–0.977
**Gender**				
Male	*REF*		*REF*	
Female	1.16 *	1.09–1.25	1.04	1.00–1.14
Transgender	2.29 *	1.59–3.23	1.59 *	1.07–2.33
None of these	1.57 *	1.20–2.04	1.90 *	1.41–2.52
**Marital status**				
Now married	*REF*		*REF*	
Widowed	1.26	1.05–1.22	1.45 *	1.15–1.81
Divorced	1.25 *	1.14–1.38	1.23 *	1.09–1.38
Separated	1.56	1.24–1.95	1.57 *	1.21–2.03
Never married	1.33 *	1.22–1.44	1.32 *	1.19–1.46
**Work for pay in the last 7 days**				
Yes	*REF*		*REF*	
No	1.13 *	1.05–1.22	1.26 *	1.15–1.39
**Difficulty paying for expenses in the last 7 days**				
Not at all difficult	*REF*		*REF*	
A little difficult	1.64 *	1.49–1.81	1.69 *	1.46–1.95
Somewhat difficult	2.92 *	2.64–3.24	2.83 *	2.44–3.28
Very difficult	6.53 *	5.82–7.34	7.05 *	6.05–8.23
**Food insecure ^a^**				
No	*REF*		*REF*	
Yes	1.50 *	1.39–1.63	1.57 *	1.42–1.74
**Housing insecure ^b^**				
No	*REF*		*REF*	
Yes	1.19 *	1.05–1.34	1.23 *	1.08–1.40

* *p* < 0.05. ^a^ Food insecurity was defined as participants reporting that they had enough to eat but not always the kinds of food I/we wanted to eat, sometimes not enough to eat, or often not enough to eat. ^b^ Housing insecure was defined as either rent/mortgage not being current or eviction/foreclosure being somewhat/very likely.

**Table 4:** Interaction model.

	OR (Odds of Having Severe Psychological Distress)	95% Confidence Interval
**Disability**		
No	*REF*	
Yes	5.60 *	4.27–7.45
**Age**	0.97 *	**0.97–0.977**
**Gender**		
Male	*REF*	
Female	1.04	0.95–1.14
Transgender	1.59 *	1.07–2.34
None of these	1.89 *	1.41–2.52
**Marital Status**		
Now married	*REF*	
Widowed	1.45 *	1.15–1.81
Divorced	1.23 *	1.09–1.38
Separated	1.57 *	1.21–2.03
Never married	1.32 *	1.19–1.46
**Work for pay in the last 7 days**		
Yes	*REF*	
No	1.26 *	1.15–1.39
**Difficulty paying for expenses in the past 7 days**		
Not at all difficult	*REF*	
A little difficult	2.29 *	1.63–3.22
Somewhat difficult	3.26 *	2.29–4.64
Very difficult	9.81 *	7.11–13.64
Difficulty paying for expenses in the past 7 days * disability		
Not at all difficult	*REF*	
A little difficult	0.69 *	0.47–0.99
Somewhat difficult	0.83	0.56–1.21
Very difficult	0.67 *	0.47–0.94
**Food insecurity ^a^**		
No	*REF*	
Yes	1.57 *	1.42–1.74
**Housing insecurity ^b^**		
No	*REF*	
Yes	1.23 *	1.08–1.41

* *p* < 0.05. ^a^ Food insecurity was defined as participants reporting that they had enough to eat but not always the kinds of food I/we wanted to eat, sometimes not enough to eat, or often not enough to eat. ^b^ Housing insecure was defined as either rent/mortgage not being current or eviction/foreclosure being somewhat/very likely.

**Table 5:** Odds of moderate and severe psychological distress (univariate associations).

	Odds Ratio (Odds of Moderate Psychological Distress)	95% Confidence Interval	Odds Ratio (Odds of Severe Psychological Distress)	95% Confidence Interval
**Age (years)**				
	0.97 *	0.97–0.97	0.97 *	0.97–0.97
**Hispanic, Latino, or Spanish origin**				
No	REF	REF	REF	REF
Yes	1.27 *	1.13–1.42	1.14	0.99–1.31
**Race**				
White alone	REF	REF	REF	REF
Black alone	1.06	0.93–1.20	1.08	0.92–1.25
Asian alone	0.92	0.76–1.11	0.87	0.68–1.09
Any other race alone or race in combination	1.54 *	1.35–1.75	1.44 *	1.23–1.68
**Highest degree or level of school completed**				
Less than high school	REF	REF	REF	REF
Some high school	1.05	0.63–1.76	0.97	0.54–1.81
High school graduate or equivalent	0.83	0.53–1.31	0.88	0.53–1.52
Some college, no degree/in progress	0.84	0.54–1.32	0.85	0.52–1.47
Associate’s degree (e.g., AA, AS)	0.81	0.52–1.27	0.81	0.49–1.40
Bachelor’s degree (e.g., BA, BS, AB)	0.59	0.38–0.93	0.58	0.35–1.00
Graduate degree (e.g., master’s, professional, doctorate)	0.49	0.31–0.77	0.47 *	0.28–0.81
**Marital status**				
Now married	REF	REF	REF	REF
Widowed	1.01	0.85–1.20	1.17	0.94–1.44
Divorced	1.41 *	1.29–1.55	1.39	1.24–1.56
Separated	2.53 *	2.02–3.16	2.78 *	2.16–3.60
Never married	2.13 *	1.97–2.31	2.06 *	1.87–2.27
**Gender**				
Male	REF	REF	REF	REF
Female	1.36 *	1.26–1.46	1.26 *	1.15–1.38
Transgender	4.14 *	2.93–5.90	3.13 *	2.17–4.47
None of these	2.41 *	1.85–3.13	2.91 *	2.19–3.84
**2021 household income before taxes**				
Less than USD 25,000	3.77 *	3.19–4.48	4.41 *	3.51–5.60
USD 25,000–34,999	2.81 *	2.35–3.37	3.30 *	2.59–4.23
USD 35,000–49,999	2.70 *	2.27–3.22	3.00 *	2.37–3.84
USD 50,000–74,999	2.30 *	1.95–2.27	2.60 *	2.07–3.31
USD 75,000–99,999	1.82 *	1.54–2.17	2.04 *	1.61–2.62
USD 100,000–149,999	1.34 *	1.13–1.60	1.50 *	1.18–1.92
USD 150,000–199,999	1.12 *	0.92–1.37	1.10	0.82–1.47
USD 200,000 and above	REF	REF	REF	REF
**Covered by public or private insurance**				
Yes	REF	REF	REF	REF
No	2.31 *	2.01–2.64	2.30 *	1.98–2.66
**Work for either pay or profit in the last 7 days**				
Yes	REF	REF	REF	REF
No	0.99	0.92–1.06	1.14 *	1.04–1.24
Not working (do not want to work) ^a^	0.44 *	0.40–0.49	0.42 *	0.36–0.48
Not working (not able to work)	1.75 *	1.61–1.91	1.98 *	1.80–2.18
**Difficulty paying usual household expenses**				
Not at all difficult	REF	REF	REF	REF
A little difficult	1.90 *	1.71–2.12	1.98 *	1.70–2.32
Somewhat difficult	3.89 *	3.51–4.31	3.96 *	3.42–4.59
Very difficult	10.31 *	9.25–11.49	11.63 *	10.12–13.41
**Food insecurity ^b^**				
Food insecure	3.37 *	3.14–3.61	3.75 *	3.43–4.10
Not food insecure	REF	REF	REF	REF
Enough of the kinds of food I/we wanted to eat	REF	REF	REF	REF
Enough, but not always the kinds of food I/we wanted to eat	2.77 *	2.57–2.98	2.87 *	2.60–3.16
Sometimes not enough to eat	5.28 *	4.67–5.97	6.07 *	5.31–6.94
Often not enough to eat	11.29 *	8.99–14.30	14.3 *	11.58–17.69
**Free groceries/meals or Supplemental Nutrition Assistance Program (SNAP) within the last 7 days**				
Did not receive SNAP and/or other free groceries/meals	REF	REF	REF	REF
Received SNAP or other free groceries/meals	1.95 *	1.78–2.13	2.12 *	1.91–2.35
**Free groceries from a food pantry, food bank, church, or other place that helps with free food**				
Yes	REF	REF	REF	REF
No	1.92 *	1.67–2.21	2.06 *	1.76–2.40
**Received benefits from the Supplemental Nutrition Assistance Program (SNAP) within the last 7 days**				
Yes	REF	REF	REF	REF
No	1.96 *	1.77–2.16	2.09 *	1.87–2.35
**Housing insecurity ^c^**				
Housing insecure	2.95 *	2.63–3.32	3.17 *	2.80–3.58
Not housing insecure	REF	REF	REF	REF

* = *p* < 0.01. ^a^ Defined as not wanting to work: did not want to be employed at this time or retired. ^b^ Food insecurity was defined as participants reporting that they had enough to eat but not always the kinds of food I/we wanted to eat, sometimes not enough to eat, or often not enough to eat. ^c^ Housing insecure was defined as either rent/mortgage not being current or eviction/foreclosure being somewhat/very likely.

## Data Availability

This dataset is publicly available from the United States Census Bureau. It can be accessed at: https://www.census.gov/programs-surveys/household-pulse-survey/data/datasets.2022.html#list-tab-1264157801 (accessed on 5 August 2024).
